# Socio-Organizational Impact of Confocal Laser Endomicroscopy in Neurosurgery and Neuropathology: Results from a Process Analysis and Expert Survey

**DOI:** 10.3390/diagnostics11112128

**Published:** 2021-11-16

**Authors:** Marina L. Fotteler, Friederike Liesche-Starnecker, Maria C. Brielmaier, Johannes Schobel, Jens Gempt, Jürgen Schlegel, Walter Swoboda

**Affiliations:** 1 DigiHealth Institute, Neu-Ulm University of Applied Sciences, 89231 Neu-Ulm, Germany; johannes.schobel@hnu.de (J.S.); walter.swoboda@hnu.de (W.S.); 2 Institute for Geriatric Research at Agaplesion Bethesda Ulm, Ulm University, 89073 Ulm, Germany; 3 Department of Neuropathology, Institute of Pathology, School of Medicine, Technical University Munich, 81675 Munich, Germany; friederike.liesche@tum.de (F.L.-S.); maria.brielmaier@gmx.de (M.C.B.); schlegel@tum.de (J.S.); 4 Department of Neurosurgery, School of Medicine, Klinikum Rechts der Isar, Technical University Munich, 81675 Munich, Germany; jens.gempt@tum.de

**Keywords:** confocal laser endomicroscopy, neurosurgery, neuropathology, frozen sections, healthcare processes, clinical workflow analysis

## Abstract

During brain tumor resection surgery, it is essential to determine the tumor borders as the extent of resection is important for post-operative patient survival. The current process of removing a tissue sample for frozen section analysis has several shortcomings that might be overcome by confocal laser endomicroscopy (CLE). CLE is a promising new technology enabling the digital in vivo visualization of tissue structures in near real-time. Research on the socio-organizational impact of introducing this new methodology to routine care in neurosurgery and neuropathology is scarce. We analyzed a potential clinical workflow employing CLE by comparing it to the current process. Additionally, a small expert survey was conducted to collect data on the opinion of clinical staff working with CLE. While CLE can contribute to a workload reduction for neuropathologists and enable a shorter process and a more efficient use of resources, the effort for neurosurgeons and surgery assistants might increase. Experts agree that CLE offers huge potential for better diagnosis and therapy but also see challenges, especially due to the current state of experimental use, including a risk for misinterpretations and the need for special training. Future studies will show whether CLE can become part of routine care.

## 1. Introduction

In brain tumor surgery, the extent of resection is essential for post-operative patient recovery and survival [1,2,3]. To keep the damage of healthy tissue to a minimum, tumor borders need to be determined intraoperatively [4]. Usually, macro- or microscopic tissue analysis is needed to reliably assess the tissue status [2,5]. Currently, frozen sections are the state-of-the-art procedure to determine tumor borders and type. A biopsy is removed by the neurosurgeon and sent to the neuropathologist for histopathological frozen section analysis.

However, the described process has several disadvantages. As it is an invasive method, usually only one sample is removed and analyzed. Additionally, damage to the brain tissue is possible. Preparing and freezing the tissue sample in the lab can change or destroy structures which are important for diagnosis. Additionally, it can take up to 45 min until the results are ready and can be communicated to the neurosurgeon [2,4,6].

The last couple of years have seen the emergence of a new technology that has the potential to digitize and improve intraoperative tissue analysis and decision making. Confocal laser endomicroscopy (CLE) is a promising digital biopsy approach using a high-resolution microscope located in a handheld probe. By placing the probe directly on the surgical site, tissue structures can be visualized in vivo in near real-time [7]. The procedure is non-destructive, allowing the investigation of several tumor areas. Combined with a fluorescent agent administered prior to using the device, CLE enables tissue differentiation, tumor type diagnosis, and the identification of tumor borders comparable to frozen sections. This has been shown in several studies, both for in vivo and ex vivo analyses of animal and human brain tumor tissue [1,8,9,10,11]. The authors of a recent systematic literature review, however, found the evidence-base to be insufficient to formulate a definite conclusion on the usefulness of CLE for intraoperative decision support [12]. Yet, several other medical fields such as gastroenterology [13,14], urology [15], and others [5,16,17] report promising results for the effective and safe use of CLE.

Several CLE devices from different manufacturers and optimized for different medical fields are available on the market. In neurosurgery, the technology is still in an experimental phase and not yet part of the current treatment regime. In order to introduce this promising technology into daily routine, it is essential to properly understand the underlying clinical processes [18]. As stated by Ammenwerth and Hackl [18], the impact on socio-organizational structures within the institution is especially important. Therefore, the connection between involved personnel, necessary activities, as well as the required infrastructure must be clarified. To that extent, process modelling is a well-established methodology providing a (graphical) language that can be understood by both computer scientists and physicians [19,20]. To our knowledge, no study explicitly addressed socio-organizational aspects of the introduction of CLE into routine neurosurgical diagnostic and therapy so far. A recently published conceptual model on the impact of CLE included three hypotheses focusing on socio-economic aspects, specifically, the increase in the workload for clinical staff, the high cost, and the potentially longer process duration overall due to the maintenance of the device [21]. The aim of this study was to answer the following two research questions (RQ) using the case example of brain tumor resection surgery:

RQ1: What could a workflow employing CLE in neurosurgery look like compared to the current clinical process analyzing frozen sections?

RQ2: What are the opinions of clinical staff working with CLE regarding its socio-organizational aspects?

## 2. Methods

To investigate the potential of CLE for neurosurgery and neuropathology and answer the research questions, we (1) modelled and analyzed the current process of frozen sections, (2) modelled a digital process visualizing the possible procedure with CLE, (3) compared both process models, and (4) conducted a survey among medical experts working with the new technology. Our study was conducted based on the ZEISS CONVIVO^®^, a second-generation CLE device optimized for neurosurgery that has been on the market since 2018. The intraoperative use of this device has been described in recent studies [11,22]. In 2020, a digital platform for neuropathologists has been released allowing the expert to remotely diagnose images instead of being available in the operating theatre. The CLE model presented in this study includes this new In Vivo Pathology Suite [23].

### 2.1. Process Analysis

The case of surgical brain tumor resection has been used as the basis for the process analysis. Both processes (current and CLE) were modelled using Business Process Model and Notation (BPMN 2.0), a high-level standard used to graphically document the steps in a workflow [24]. BPMN is mathematically complete and particularly useful to visualize and understand complex processes [18]. A web-based tool, Signavio Explorer Version 15.6.0 by the BPMN Academic Initiative, was used for modelling.

Data collection for the current process of tissue diagnosis using frozen sections took place in a large clinic for maximum care located in Southern Germany. To model the process, non-structured expert interviews were conducted with two neuropathologists who guided the interviewer through the clinical and laboratory process. Additionally, observations and time measurements for the biopsy resection, the transport, and the frozen section analysis were conducted on site. To collect data and model the digitized process with CLE, engineers and project managers from the manufacturer of the microscope were interviewed. Additionally, presentation material, manuals, and other documents such as visual boards depicting the envisioned workflow were consulted. The resulting CLE model shows what the process could look like once CLE has been established. After introducing the experts to the BPMN syntax, both models were refined in several cycles to ensure accuracy and completeness.

### 2.2. Expert Survey

An online questionnaire was developed consisting of 16 questions in four sections: general information, work with the CLE device, opinion of the microscope, and biggest advantages/disadvantages. The questionnaire was written in German, and a translated version can be found in Table 1. The community edition for the web-based Open-Source software LimeSurvey (version 3.x, LimeSurvey GmbH, Hamburg, Germany) was used for the online survey. The questionnaire was distributed with individual access codes to neuropathologists, neurosurgeons and medicine students working with the confocal microscope in vivo and ex vivo at three German hospitals. The survey was anonymous. All participants gave their informed consent. The survey was sent out between April and September 2021. Up to three reminders were sent. An ethics vote was obtained for the survey from the Joint Ethics Committee of the Bavarian Universities of Applied Sciences (approval code GEHBa-202008-V-008, approval date 7 October 2020).

## 3. Results

### 3.1. Process Analysis (Results for RQ1)

Two process models were created using the BPMN 2.0 notation, one depicting the current process of frozen section analysis and the other one visualizing a process using CLE. In the following section we present and describe collapsed and simplified versions of the process models. The process comparison is based on the detailed models, which can be found in the supplementary material (Figure S1: current process, Figure S2: digitized process).

#### 3.1.1. Current Process

**Figure 1:**
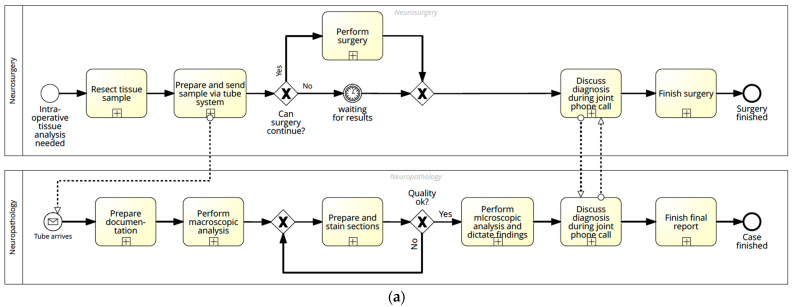

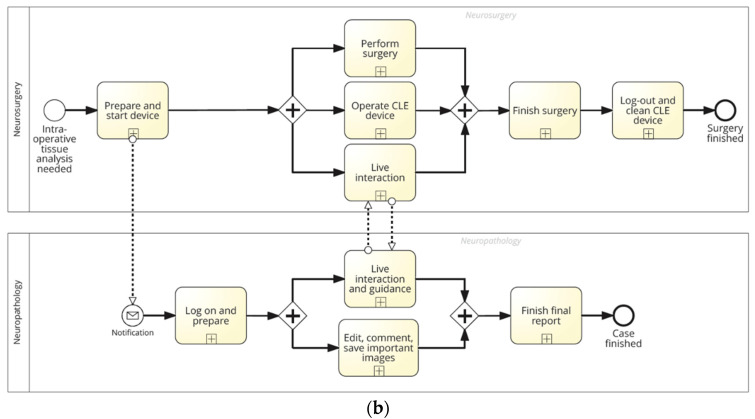
Simplified versions of the two processes: (**a**) Current process of frozen section analysis; (**b**) Digitized process using CLE. Circles mark the start and end of the process, arrows show the process flow, rectangles represent tasks, tasks with the “+” indicate collapsed subprocesses, the diamond with the “×” represents an exclusive gateway (XOR), meaning only one path continues, and the diamond with the “+” denotes a parallel gateway (AND), meaning all paths continue.

The process starts with the need for an intraoperative histology. Once the surgical site is prepared, the neurosurgeon takes a tissue sample, which is then sent to the neuropathology laboratory together with the request form including all necessary information via a tube system. At the reception, the request form is processed, and relevant information is added. A laboratory technician and the Jr. neuropathologist are informed, and the sample is transported to the laboratory. Macroscopic analysis is performed, and the first impressions are dictated by the Jr. neuropathologist, possibly supervised by a Sr. neuropathologist. After embedding and freezing, a laboratory technician prepares the frozen sections (slicing and staining with hematoxylin and eosin (HE)). After having checked the quality and potentially requested a new section, the microscopic analysis is performed by a Jr. neuropathologist alone or together with the Sr. neuropathologist. They record the results and report them back to the surgery room using a regular phone call. The final report is typed by the secretary (Figure 1a).

In addition to modelling the process, the time needed for the analysis of frozen sections was measured. Three sections were timed separately: (1) the resection and packing of the biopsy, (2) the transport to the laboratory, and (3) the preparation of sections, analysis, and report of results. On average, 4:25 min were needed to extract and pack the biopsy (four measurements ranging from 2:28 to 6:52 min). For the tube transport, three measurements were taken, resulting in an average duration of 1:40 min. Four measurements were taken for the tasks conducted in the laboratory. The average duration was 11:57 min, with a range from 08:53 to 15:04 min. On average, 18:02 min passed from biopsy resection to the reception of results in this clinic.

#### 3.1.2. CLE Process

When working with the CLE device, the surgery assistant logs into the device and establishes a connection. Then, the case is created by importing data from a radiology system (here, the DICOM Modality Worklist) or by entering the information manually. The neuropathologist or a team of neuropathologists receive a consultation request and the neuropathologist scheduled to participate in the surgery can log in to review the available patient information in an anonymized format. Once the live stream is established by the surgery assistant, different image capturing modi are available. A continuous audio communication can be made available via the traditional channels (phone, intercom). Thus, the neurosurgeon and neuropathologist are able to cooperate and jointly assess the tumor region. Images that are considered important by the neuropathologist can be edited, annotated, and saved for the final report. While the surgery continues, the neuropathologist finishes entering the diagnostic information and selecting images. The final report is created automatically and can also be accessed by the neurosurgeon. After logging off, the device is cleaned by the surgery personnel (Figure 1b).

#### 3.1.3. Process Comparison

In both processes, two parties are involved, the clinic for neurosurgery and the department of neuropathology. However, while the current process requires up to seven people (neurosurgeon, surgery assistant, neuropathology receptionist, neuropathology secretary, lab technician, Jr. neuropathologist, and Sr. neuropathologist), the digital process using the CLE device only requires three people (neurosurgeon, surgery assistant, neuropathologist). Different indicators comparing the two workflows are listed in Table 2. The number of tasks overall decreased from 42 to 26 (−38.1%). However, while tasks in the neuropathology department decreased due to the elimination of administrative and laboratory tasks from 30 to 9 (−70%), the neurosurgeon and the surgery personnel are faced with a slight increase in tasks due to the maintenance and operation of the device from 12 to 17 tasks (+41.7%). If several images are taken, which is one of the main advantages of CLE, the workload for the neurosurgery increases even further. The digitized process relies solely on digital and phone communication compared to the paper documentation in the current process.

### 3.2. Expert Survey (Results for RQ2)

Fifteen experts were contacted for the online survey of which twelve responded (80% response rate). Table 3 characterizes the study participants. The majority of participants (66.7%) were between 30–59 years of age. Seven (58.3%) were male, half worked as a neurosurgeon while a quarter each were neuropathologists and medical students. With the exception of one person, all had experience with CLE from more than five surgeries. Interestingly, no participants stated that they were dissatisfied with the device, while 10 participants (83.3%) were overall satisfied with the device and 2 (16.7%) had a neutral position.

The characteristics receiving the highest number of positive votes were usability, integration into daily routine, and patient safety (N = 10 each). The images provided by the CLE device were voted to be of medium quality by six people versus five who found them to be good. The tasks conducted with the device include patient education (N = 4), appointment planning (N = 4), preparation in the operating theatre room (N = 6), login (N = 6), preparation of the sterile scanner probe (N = 6), selection/alternation of the modus (N = 7), placement of the probe on the tissue (N = 6), interpretation of images (N = 4), editing the images (N = 4), logoff (N = 5), and cleaning (N = 2).

Five participants (41.6%; three neurosurgeons, two neuropathologists) expressed an increase in their workload due to the use of the CLE device. They explained this with the fact that the methodology is currently used as an addition to the frozen section analysis, not as a replacement. Preparation, operation, and cleaning were mentioned as other reasons. However, nine experts (75%) think that the surgery time can be reduced once the device is used in standardized clinical routine.

In general, the faster diagnosis and shortened surgery duration is the biggest advantage of CLE, mentioned by seven experts (58.3%), four of them neurosurgeons. This is followed by an improved diagnosis and resection when using the device. The training and experience needed to effectively use CLE are current disadvantages mentioned six times by neurosurgeons, neuropathologists, and medical students in even measure. The issue of user dependency was only mentioned by neurosurgeons. All advantages and disadvantages mentioned by the experts are listed in Table 4 (answers to part 4 of the questionnaire: final questions).

## 4. Discussion

To our knowledge, this is the first study investigating the impact of CLE on the clinical workflow and staff in neuropathology and neurosurgery. Other studies have cursorily discussed potential implications of CLE for workflows and staff but have not explicitly analyzed these issues [1,2,22,25].

BPMN proved to be feasible to model and compare clinical processes. A clinical workflow relying on CLE in neurosurgery and neuropathology could be successfully represented. Several advantages have been shown. Particularly, administrative and laboratory staff in the neuropathology can be relieved. Neuropathologists benefit from a streamlined workflow without interruptions and the fact that they can conduct diagnoses remotely from their desk. Additionally, a continuous communication, directly involving both experts in the diagnosis and treatment process, is possible. The neuropathologist can provide guidance while images can be analyzed in real-time (i.e., no transport via tube) [7]. The team of the neurosurgery, i.e., the neurosurgeon and any surgery assistant, face a potential increase in workload due to the preparation, operation, and cleaning procedures of the CLE device, confirming previously published hypotheses [21]. Especially when investigating multiple tumor areas, which is one of the main advantages of CLE, clinical staff are burdened with additional work.

More than 80% of the interviewed experts were satisfied with the device, and no survey participant voiced general dissatisfaction. The potential to shorten surgery time from incision to closure and improvement of resection were confirmed. Our measurements resulted in an average time of approximately 18 min for the frozen sections (including biopsy resection and transport), other authors report average durations of 30 to 45 min [2,5]. Longer distances or a lack of neuropathologists are possible aspects that can lead to longer duration. However, much of this time can be saved in a process employing CLE. A potentially shortened surgery duration is highly advantageous for the patient [3,8] and can also be economically beneficial [26]. Other advantages from a medical point of view are mainly the elimination of the need for tissue removal for the biopsy and the opportunity to analyze several tumor regions [27].

Ten experts highlighted the need for training and experience for both the neurosurgeon and the neuropathologist before reliable diagnoses can be performed [2,28]. Images produced by CLE differ from those known to neuropathologists. In this context, the lack of a reference catalogue has been mentioned. Particularly in the beginning, the interpretation of images can be challenging or imprecise, including the risk for misdiagnosis [12]. However, this is most likely only a temporary challenge. Once the technology is better established, an atlas with images will be available, and the use of CLE becomes part of medical training, this will become less and less of an issue. Other researchers mention the small field of view, motion artifacts, and image obscuring through blood cells that can inhibit the data quality. However, existing studies showed that this can be solved by cleaning the probe or irrigating the surgical field [2,3]. With the option to analyze several samples, these problems can be further mitigated. Further potential shortcomings of CLE revealed in the expert comments are the added workload caused by an additional device in the operating theatre confirming the results from the process analysis. Finally, CLE is linked to considerable costs with prices of USD 200,000 and above for the device, not including operation and maintenance [17]. The sterile sheaths, for which currently only one manufacturer exists, and the fluorescent agent cause additional costs. The sheaths cost up to EUR 40 per piece. The current contrasting agent Natriumfluorescein (NaF) costs between EUR 8–10 per vial of 500 mg. Per patient, a minimum of one vial is needed.

The use of CLE in clinical settings is still in a preliminary phase leaving many blank spots to be investigated further. Potentially groundbreaking for future clinical research and diagnostic is the chance to visualize live tumor cells with CLE. The realization of telepathology, or teleneuropathology, is another promising aspect associated with the implementation of CLE. As CLE produces digital images of tissue structures, remote analysis by neuropathologists from other clinics or laboratories is possible [10,29]. A small, highly proficient team can assist smaller institutions for the diagnosis of difficult cases. Considering the shortage of expert neuropathologists, this can improve clinical care in hospitals without a neuropathology department. All survey participants confirmed the future-oriented character of teleneuropathology. Pioneering studies also show the potential to automatically analyze CLE images using deep learning methods [30,31,32].

This study has limitations. The experimental nature of CLE implementation limits the applicability of the results from this study. The current process for the analysis of frozen sections modelled here is based on the workflow in a German clinic. Even though the analysis of frozen sections is a standardized routine, the results might not be generalizable to other countries. The CLE device is stored in another room and needs to be transported to the operating theatre before performing the surgery and back again after surgery. These tasks are not represented in the model as both processes start with the intraoperative histopathological analysis to limit complexity. Additionally, an anesthetist administers NaF directly after the first incision. Albeit important for the tissue analysis, this task is negligible regarding the overall process complexity as the anesthetist is present during the entire surgery to monitor vital signs and narcosis. CLE is not yet integrated into standardized care, therefore the number of experts with experience using the methodology is limited. The results of the small sample included in this survey lack reliability. In light of other positive research results regarding the use of CLE in neuropathology and neurosurgery, this study provides additional confirmation for the potential of this methodology and presents a first insight into the socio-organizational consequences of CLE. Additionally, it can serve as the basis for future research, e.g., a thorough economic evaluation of CLE in the form of a dynamic investment calculation for the setting of neuropathology and neurosurgery.

## 5. Conclusions

This study confirms the potential of CLE for the improvement of intraoperative tissue analysis and decision making in neurosurgery, specifically during brain tumor resection. The streamlining of processes, faster and better diagnosis and tumor resection, and workload relief for neuropathologists are some of the main advantages. Training experts to interpret CLE images will be of the utmost importance for an establishment of CLE. The results of future studies, in particular economic evaluation and larger clinical trials, will determine whether the technology can become part of standardized care. Many possible applications of CLE in clinical care have yet to be studied. The visualization and analysis of live cancer cells, telepathology, and the combination with deep learning methods hold large promise for the safe, effective, and efficient delivery of care in the future.

## Figures and Tables

**Table 1:** List of all survey questions.

Section	Questions
1. General information	How old are you? What is your gender? What is your position? How long have you been working in this position?
2. Your work with the device	Conditional question depending on position: How many operations have you conducted/assisted with using the device?/What is the number of operations using the device you analyzed images of? ^1^ What tasks do you conduct with the device? (multiple choice) How does your workload change when using the device? Conditional question: Please explain how your workload increased/decreased. ^1^
3. Your opinion of the device	How do you rate the following indicators on a scale from 1–3: usability, integration, reliability, data/image quality, medicinal use, patient safety, data protection? Do you think it makes sense to use the device for standard diagnostics? Conditional question: Please comment why you do not think that this makes sense. ^1^ Do you think, surgery duration could be shortened using the device? What is your opinion on telepathology? How satisfied are you overall with the device?
4. Final questions	What do you think are the biggest advantages/chances of the device? What do you think are the biggest disadvantages/risks of the device?

^1^ Previous answer determined phrasing or appearance of this question.

**Table 2:** Comparison of the current process and the CLE process.

	Current Process	CLE Process
Parties	2	2
Staff members	6–7	3
Tasks total	42	26
Tasks neurosurgery	12	17
Tasks neuropathology	30	9
Transfers between parties	2	2
Medium of transfer	tube (paper, sample), phone	digital, phone
Time until results are ready	~18:02 min	immediately (continuous communication)

**Table 3:** Characteristics of the survey participants.

	Total	Satisfaction with CLE Device (N) ^1^	
	N (%)	Satisfied	Neutral
**Age**			
Below 30 years	3 (25.0)	3	0
30–59 years	8 (66.7)	6	2
60 years or older	1 (8.3)	1	0
**Gender**			
Female	5 (41.7)	4	1
Male	7 (58.3)	6	1
**Position**			
Neurosurgeon	6 (50.0)	5	1
Neuropathologist	3 (25.0)	2	1
Medical student	3 (25.0)	3	0
**Experience**			
<5 years	3 (25.0)	3	0
5–10 years	5 (41.7)	4	1
>10 years	4 (33.3)	3	1
**Number of operations with CLE**			
<5	1 (8.3)	0	1
5–20	4 (33.3)	4	0
>20	7 (58.3)	6	1

^1^ The option ‘dissatisfied’ was not selected by the participants. For brevity purposes, the table only shows the distribution between the options ‘satisfied’ and ‘neutral’.

**Table 4:** Advantages and disadvantages of CLE according to the survey participants (frequency).

Advantages	Disadvantages
- Faster diagnosis (real-time) and surgery (N *=* 7) - Better diagnosis and resection (N *=* 6) - Potential for future progress (e.g., visualization of live tissue, use of artificial intelligence) (N *=* 5) - Unlimited biopsies (non-destructive) (N *=* 3) - Remote analysis (N *=* 1)	Training and experience needed (N *=* 6) Imprecise diagnosis, risk of misinterpretation (N *=* 4) User dependent (N *=* 2) Additional effort for neurosurgery (N *=* 2) Price (N *=* 1)

## Data Availability

All data collected for the process models have been incorporated into the respective BPMN models, which can be found in the supplementary material. The data from the survey were collected in German and translated for this manuscript. The data can be made available upon request.

## Supplementary Materials

The following are available online at www.mdpi.com/article/10.3390/diagnostics11112128/s1, Figure S1: Full BPMN model for the current process of frozen section analysis in a large German clinic for maximum care, Figure S2: Full BPMN model for the digitized process with the CLE device ZEISS CONVIVO^®^ by the Carl Zeiss Meditech AG.
