# Supplementary material for: Socio-Organizational Impact of Confocal Laser Endomicroscopy in Neurosurgery and Neuropathology: Results from a Process Analysis and Expert Survey
Source: Diagnostics (Basel). 2021 Nov 16;11(11):2128. doi: 10.3390/diagnostics11112128 (PMC8623423; doi:10.3390/diagnostics11112128)
Supplement: Supplementary file 1 [file diagnostics-11-02128-s001.zip › Figure S2 Full BPMN model for the digitized process with CLE.pdf]

Figure S2: Full BPMN model for the digitized process with CLE

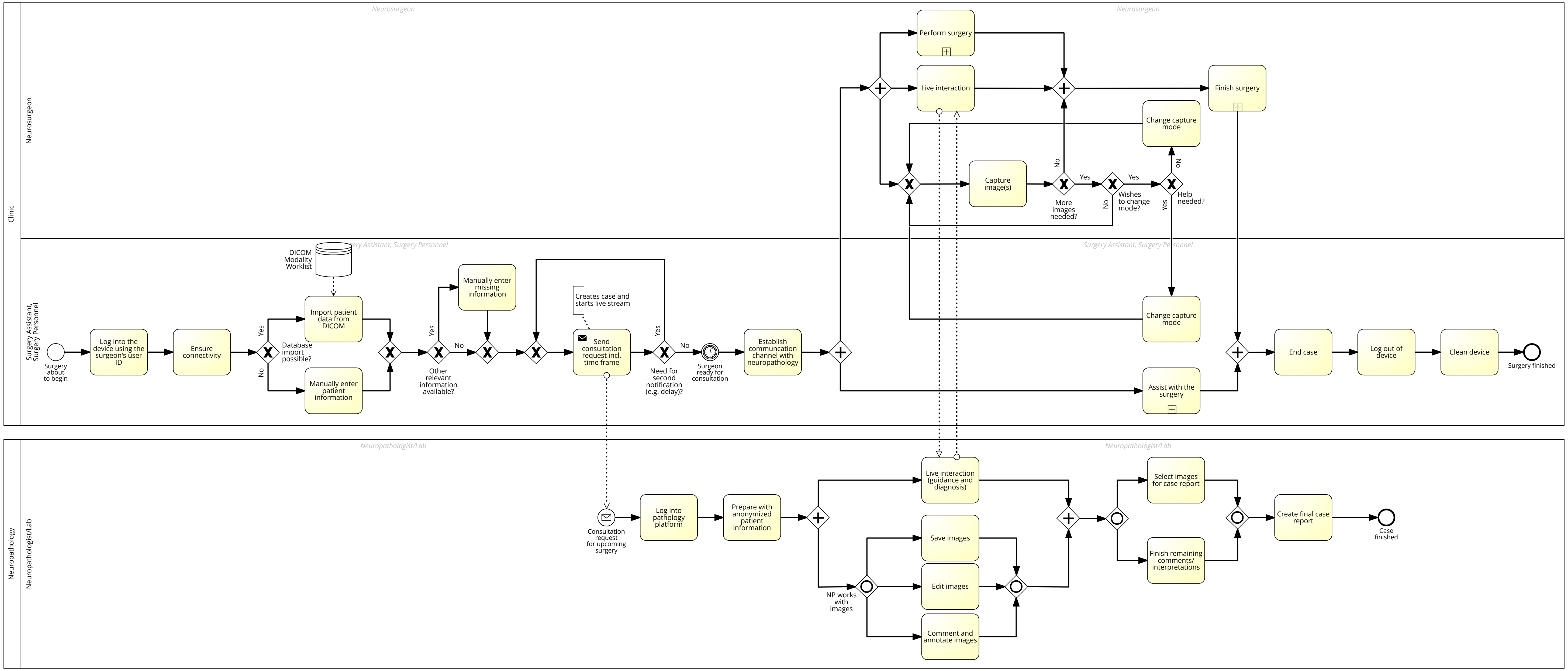

BPMN: Business Process Model and Notation
CLE: Confocal Laser Endomicroscopy
DICOM: Digital Imaging and Communications in Medicine (data standard for medical imaging data)
