# Supplementary material for: Socio-Organizational Impact of Confocal Laser Endomicroscopy in Neurosurgery and Neuropathology: Results from a Process Analysis and Expert Survey
Source: Diagnostics (Basel). 2021 Nov 16;11(11):2128. doi: 10.3390/diagnostics11112128 (PMC8623423; doi:10.3390/diagnostics11112128)
Supplement: Supplementary file 1 [file diagnostics-11-02128-s001.zip › Figure S1 Full BPMN model for the current process of frozen section analysis in a large German clinic for maximum care.pdf]

Figure S1: Full BPMN model for the current process of frozen section analysis in a large German clinic for maximum care

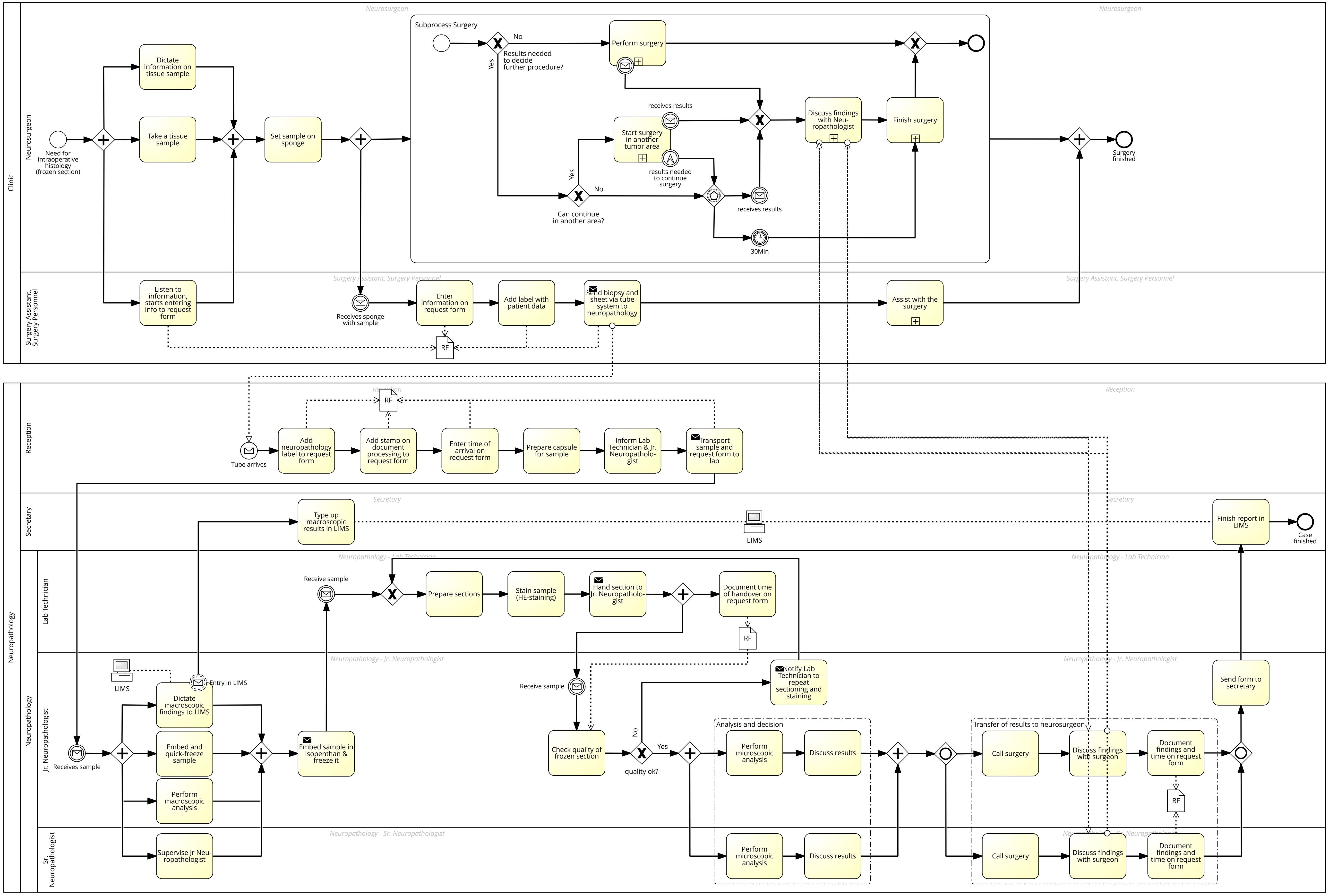

BPMN: Business Process Model and Notation
RF: Request Form
LIMS: Laboratory Information System
HE: Hematoxylin and eosin (used to stain frozen section)
